# Self-Supervised Multi-Task Learning for the Detection and Classification of RHD-Induced Valvular Pathology

**DOI:** 10.3390/jimaging11040097

**Published:** 2025-03-25

**Authors:** Lorna Mugambi, Ciira wa Maina, Liesl Zühlke

**Affiliations:** 1 Centre for Data Science and Artificial Intelligence, Dedan Kimathi University of Technology, Nyeri 10143, Kenya; 2 South African Medical Research Council, Francie Van Zyl Drive, Cape Town 7505, South Africa; 3 Division of Paediatric Cardiology, Department of Paediatrics and Child Health, Red Cross War Memorial Children’s Hospital, Cape Town 7700, South Africa; 4 Division of Cardiology, Department of Medicine, Groote Schuur Hospital, Cape Town 7925, South Africa

**Keywords:** clustering, echocardiography, embeddings, multi-task learning, valvular pathology, self-supervised learning

## Abstract

Rheumatic heart disease (RHD) poses a significant global health challenge, necessitating improved diagnostic tools. This study investigated the use of self-supervised multi-task learning for automated echocardiographic analysis, aiming to predict echocardiographic views, diagnose RHD conditions, and determine severity. We compared two prominent self-supervised learning (SSL) methods: DINOv2, a vision-transformer-based approach known for capturing implicit features, and simple contrastive learning representation (SimCLR), a ResNet-based contrastive learning method recognised for its simplicity and effectiveness. Both models were pre-trained on a large, unlabelled echocardiogram dataset and fine-tuned on a smaller, labelled subset. DINOv2 achieved accuracies of 92% for view classification, 98% for condition detection, and 99% for severity assessment. SimCLR demonstrated good performance as well, achieving accuracies of 99% for view classification, 92% for condition detection, and 96% for severity assessment. Embedding visualisations, using both Uniform Manifold Approximation Projection (UMAP) and t-distributed Stochastic Neighbor Embedding (t-SNE), revealed distinct clusters for all tasks in both models, indicating the effective capture of the discriminative features of the echocardiograms. This study demonstrates the potential of using self-supervised multi-task learning for automated echocardiogram analysis, offering a scalable and efficient approach to improving RHD diagnosis, especially in resource-limited settings.

## 1. Introduction

Rheumatic heart disease (RHD) is a major cardiovascular disease that primarily affects children and young adults [1,2]. While largely preventable through secondary antibiotic prophylaxis (SAP), the disease’s global prevalence has risen significantly, with Africa and South Asia bearing the heaviest burden. Recent research has improved our understanding of its natural history and management strategies, including the effectiveness of SAP [1,3]. Low- and middle-income countries (LMICs) are mostly affected by RHD, with the prevalence 80 times higher than in high-income countries. This disparity stems from low parental awareness (15%) and limited access to specialised care, leading to inadequate treatment and prevention, ultimately contributing to significant mortality and disability [4].

Group A streptococcus (GAS) infections, including both pharyngitis and skin infections in children, significantly contribute to the risk of developing acute rheumatic fever (ARF), which can progress to RHD [5]. GAS infections manifest as streptococcal pharyngitis and scarlet fever, among others, which have increased in incidence in recent years. East Africa faces a particularly significant public health challenge due to this disease, with estimates suggesting over 1.11 million children suffer from latent or confirmed RHD [6]. Socioeconomic factors such as poverty, overcrowding, and inadequate housing exacerbate the disease’s impact, increasing the risk of GAS infections and their subsequent development. Inconsistent reports on its prevalence in the region highlight the urgent need for comprehensive epidemiological studies to assess and address this burden accurately [7].

Community education about RHD and its risk factors can promote early detection and prevention. Improving access to healthcare services for the early diagnosis and management of streptococcal infections can prevent rheumatic fever, a precursor to the disease. Early detection through echocardiography is crucial, as it is more effective than heart murmurs [3,7]. By implementing these strategies, we can work towards a future where RHD is no longer a major health threat, particularly in vulnerable populations.

The World Heart Federation (WHF) published diagnostic criteria for RHD in 2012 [8]; these have been widely adopted for both population screening and clinical diagnosis. These 2012 criteria are primarily based on echocardiographic findings, which provide detailed information about the heart’s structure and function. Key principles of the 2012 WHF guidelines include:

- a heavy reliance on echocardiographic features for diagnosis;
- a distinction between “definite” and “borderline” RHD to allow for more nuanced assessments;
- a focus on abnormalities of the mitral and aortic valves, which are most commonly affected by RHD;
- and the use of standardized measurements and criteria to enhance consistency in diagnosis.

For ‘definite’ RHD in the 2012 WHF criteria, pathological mitral valve regurgitation (MR) is characterized by the presence of at least mild MR, according to some Doppler criteria, and morphological features of RHD. The morphological features of RHD include leaflet thickening, restricted leaflet motion, or chordal thickening/shortening. Similarly, ‘definite’ RHD also includes pathological aortic valve regurgitation (AR), which stems from the presence of at least mild AR with evidence of rheumatic morphological alterations, i.e., leaflet thickening/retraction and restricted motion. Mitral stenosis is also ‘definite’ RHD if there is mitral leaflet thickening, and there may be restricted valve motion [8]. Where the major criteria for ‘definite’ RHD are not met, minor criteria are used to classify cases as “borderline” RHD. These include mild MR or mild AR that does not meet the criteria for definite RHD and mild mitral or aortic valve thickening. There may also be other echocardiographic signs that are indicative of RHD, such as a dilated left atrium, evidence of RHD in other valves, or impaired left ventricular function. The echocardiographic findings must always be interpreted in the context of the patient’s clinical history and an examination, and alternative causes of the valve problems must be excluded. Moreover, the implications of these findings may vary according to the age of the patient, since some valve disease may be seen in older patients without RHD [8].

While universally applied, these 2012 guidelines had restricted practical applications. The WHF therefore updated the guidelines in 2023 to focus on strengthening patient diagnosis and the treatment of RHD. The updated 2023 WHF guidelines aim to unify disease definitions, diagnostic criteria, and management recommendations for various disease severities according to evidence-based practice [9]. A two-step screening process was established to assist in population-based screening, allowing non-experts to conduct initial screenings. Confirmatory echocardiograms are recommended for individuals with positive screening results or those without a history of ARF or RHD. The guidelines also introduced a stage-based classification system that reflects the continuous spectrums and their risk of progression, moving away from the discrete classification used in previous guidelines.

Diagnosis and management heavily rely on echocardiography, particularly in resource-limited settings where access to advanced cardiac imaging modalities may be restricted. Echocardiography provides vital information about cardiac structure and function, but its interpretation requires significant expertise and experience. In many regions where RHD is prevalent, there is a shortage of qualified healthcare professionals who can accurately interpret these images, creating a critical healthcare gap [10].

The automated analysis of echocardiogram views presents several unique challenges. Echocardiographic images often suffer from noise, varying image quality, and different acquisition protocols. Moreover, the dynamic nature of cardiac imaging means that the same structure can appear differently depending on the precise timing within the cardiac cycle. These challenges are compounded by the limited availability of labelled training data, as expert annotation is both time-consuming and expensive.

Artificial intelligence (AI) has emerged as a promising tool for enhancing the diagnosis of RHD. AI-powered systems can automate the analysis of echocardiograms, accurately detecting key indicators such as MR and classifying echocardiographic views [11,12,13]. This automation improves diagnostic accuracy and increases efficiency, particularly in resource-limited settings. Furthermore, the integration of advanced techniques including deep learning and self-supervised learning has the potential to further enhance the capabilities of AI in RHD diagnosis by enabling the interpretation of complex image data and reducing reliance on extensive labelled datasets. Among the various echocardiographic views, the parasternal long-axis (PLAX) view has been identified as the most effective for obtaining diagnostic results in screenings with AI assistance [11]. Overall, the incorporation of AI in RHD diagnosis holds great promise for improving early detection and timely intervention, ultimately leading to better patient outcomes.

Self-supervised learning (SSL) has emerged as a pivotal technique in medical imaging, particularly in echocardiography, addressing the challenge of limited labelled datasets, which is a significant barrier in medical diagnostics [14,15]. Recent advancements in SSL have demonstrated its ability to improve diagnostic accuracy and efficiency for cardiac abnormalities and valvular pathologies in echocardiograms. For instance, a study proposed a model integrating recurrent neural networks with autoencoders, achieving superior performance compared to traditional methods by enhancing feature extraction and reducing training times, showcasing SSL’s potential to improve generalization across diverse datasets [16,17]. In transthoracic echocardiography (TTE), a two-step SSL approach—pretraining on unlabelled data followed by fine-tuning on small labelled datasets—has been effective for conditions including left ventricular hypertrophy (LVH) and aortic stenosis (AS), highlighting the importance of data quality enhancement and addressing data scarcity challenges [15,18,19].

SSL’s application extends beyond echocardiography to other bio-signals, such as electrocardiography (ECG) and photoplethysmography (PPG), where traditional supervised methods struggle due to limited labelled data. SSL techniques leverage the inherent structure and temporal dependencies of these signals, employing tasks such as predicting time intervals to train models without explicit labels. This approach has shown promise in clinical applications, including the detection of arrhythmia and heart-rate variability analysis [15,20]. In magnetic resonance imaging (MRI) segmentation, SSL has addressed data scarcity by utilizing advanced algorithms that require minimal annotated data, demonstrating the effectiveness of SSL, generative models, and few-shot learning techniques across medical domains [20].

Innovative models such as EchoFM, which uses a transformer to analyse all available TTE frames, have further illustrated SSL’s potential in improving diagnostic efficiency. EchoFM outperforms existing convolutional neural networks in detecting pulmonary hypertension (PH), reducing operator variability and enhancing feature extraction [21,22]. Similarly, a multi-view video contrastive network for diagnosing aortic valve regurgitation (AR) employs supervised contrastive learning to address class imbalance and provide quick, accurate assessments of AR severity, serving as a potential pre-screening tool [23].

Despite these advancements, challenges remain in generalizing SSL methods across diverse patient populations and imaging conditions. A systematic review of contrastive learning in medical time-series data highlights the issue of limited sample annotation, which is often a time-consuming and expert-dependent task. However, contrastive learning offers a solution by learning from positive and negative samples without explicit labels, alleviating label scarcity challenges [24]. Models such as ECG-MAE, which use SSL for 12-lead ECG classification, demonstrate improved performance metrics and label efficiency, underscoring SSL’s importance in diagnosing rare diseases [25,26].

Among SSL techniques, SimCLR and DINOv2 have shown significant promise. SimCLR maximizes agreement between augmented views of the same image while minimizing agreement between views of different images, learning robust feature representations [27]. DINOv2 employs a self-distillation mechanism with a teacher–student architecture, capturing nuanced data features and improving generalization [28]. While SimCLR is widely recognized for its simplicity and effectiveness in learning discriminative features, DINOv2’s self-distillation approach offers unique advantages in capturing fine-grained details, making it suitable for medical imaging tasks where precision is critical. Recent SSL methods, such as masked autoencoders (MAE) [29] and joint self-supervised learning approaches [3], have also demonstrated strong performance in medical imaging, suggesting the need for broader comparisons to establish the proposed framework’s superiority.

This study aimed to evaluate and compare the efficacy of the SimCLR and DINOv2 self-supervised learning frameworks for the multi-task classification of echocardiographic images. The classification tasks included echocardiographic view identification, RHD condition diagnosis, and RHD severity assessment. We hypothesized that DINOv2 would outperform SimCLR in terms of classification accuracy, embedding quality and generalization capability. This was based on DINOv2’s use of a self-distillation mechanism, which encourages the model to learn robust and consistent feature representations. Additionally, DINOV2’s vision transformer architecture is known for capturing global relationships in images, whereas SimCLR relies on a CNN backbone, which primarily focuses on local patterns. Given the complex and variable nature of echocardiograms, we anticipated that DINOv2’s global perspective would prove advantageous.

## 2. Materials and Methods

This project addressed the challenge of developing a robust and data-efficient model for the multitask classification of echocardiographic images. This objective was achieved using a two-phase self-supervised learning strategy. First, SimCLR and DINOv2 were pre-trained on a large unlabelled echocardiographic dataset to learn meaningful feature representations. Second, these pre-trained models were fine-tuned on a smaller, labelled dataset within a multi-task learning framework to simultaneously perform the three classification tasks. A key aspect of this work was the evaluation and comparison of SimCLR and DINOv2 to determine which framework was better suited to multitask classification in this domain.

### 2.1. Dataset Description

The dataset, derived from a clinical database, encompasses echocardiograms of paediatric patients in South Africa. Initially, it comprised 941 static images in JPEG format and 852 videos in MP4 format, all unlabelled. The division into labelled and labelled subsets occurred post-processing, following the use of the Echo Label application, where 21 static images and 20 videos were annotated. The labels for the 20 videos were extended to all extracted frames of these videos to maintain temporal context. This process resulted in a final dataset for training and validation, consisting of 2655 labelled images and 38,037 unlabelled images, highlighting the significant role of frame extraction in scaling the dataset. The subset details are as follows:

- Unlabelled Subset: used for self-supervised pre-training, it included 920 unlabelled static images (with resolutions from 640 × 480 to 836 × 583 pixels) and 832 videos (640 × 480 pixels, 1–4 s, 30/60 fps), yielding 37,117 frames, totalling 38,037 images.
- Labelled Subset: used for supervised fine-tuning, it comprised 21 labelled static images and 20 videos (640 × 480 pixels, ~4 s, 30/60 fps), with 2634 frames extracted, totalling 2655 images. These were annotated for view, condition, and severity.

### 2.2. Data Labelling Application

To streamline the annotation of the echocardiographic data, we developed Echo Label, a web application built using the Python programming language (version 3.12) and the Flask web framework with MySQL database integration. The application’s design and the required metadata options were directly informed by the World Heart Federation’s (WHF) 2012 criteria for diagnosing rheumatic heart disease (RHD), the standard clinical guidelines for diagnosing RHD using echocardiography.

**Figure 1:**
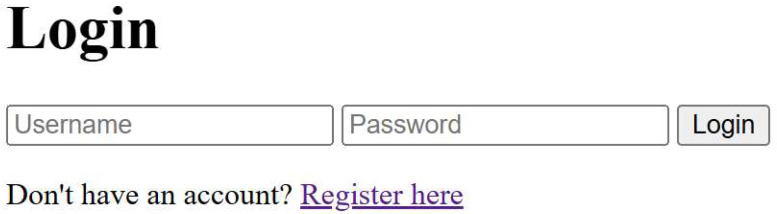
The login section of the Echo Label application.

**Figure 2:**
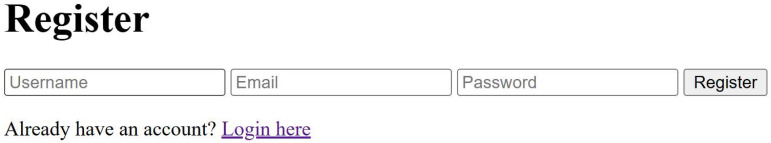
The registration section of the Echo Label application. First-time users are required to register first and then log in.

Key features of the application included:

- Secure User Authentication: Echo Label features secure login and registration functionality, ensuring that only authorized users could access and modify the data, as shown in Figure 1 and Figure 2.
- Structured Annotation Drop-Down Options (WHF 2012 Informed): the application provided a drop-down option for inputting various image attributes. It was meticulously designed to capture the key echocardiographic features emphasized by the WHF 2012 guidelines. The metadata options included:
  - Echocardiographic Views: users could select from a range of standard views, each providing a distinct perspective on cardiac anatomy and function. The options included:
    o Parasternal long axis (PLAX)
    o Parasternal short axis (PSAX)
    o Apical four chamber (A4C)
    o Subcostal
    o Suprasternal
    o Apical two chamber
    o Apical three chamber
    o Doppler
  Correctly identifying the view was essential for accurate diagnosis, as different views reveal different cardiac structures (e.g., the mitral valve is best visualized in the PLAX and A4C views).
  - Valve Thickness: users could determine the thickness of the valve leaflets, a key feature assessed in the WHF 2012 criteria. The options were:
    o Thick
    o Not thick
    o Not applicable
  - Colour: The presence of colour in an echocardiogram usually indicates the direction and speed of blood flow.
  - RHD Conditions: users could select one or more RHD-associated conditions based on the features they observe in the image where applicable. The available options included:
    o Mitral valve prolapse
    o Mitral valve regurgitation
    o Aortic valve regurgitation
    o Pulmonary valve regurgitation
    o Tricuspid valve regurgitation
    o Aortic valve stenosis
    o Mitral valve stenosis
    o Pulmonary valve stenosis
    o Tricuspid valve stenosis
    o Not applicable
  The WHF 2012 guidelines emphasize the assessment of mitral and aortic valve abnormalities, although other valves may also be affected.
  - RHD Severity: users could label the severity of RHD according to the WHF 2012 criteria. The options included:
    o Definite RHD
    o Borderline RHD
    o Normal
    o Not applicable
  - Comments: a free-text comments box was provided for each image, allowing the annotators to describe any additional observations, unusual features, or uncertainty about the assigned labels. This allowed for more nuanced information to be gathered about the images or videos.

**Figure 3:**
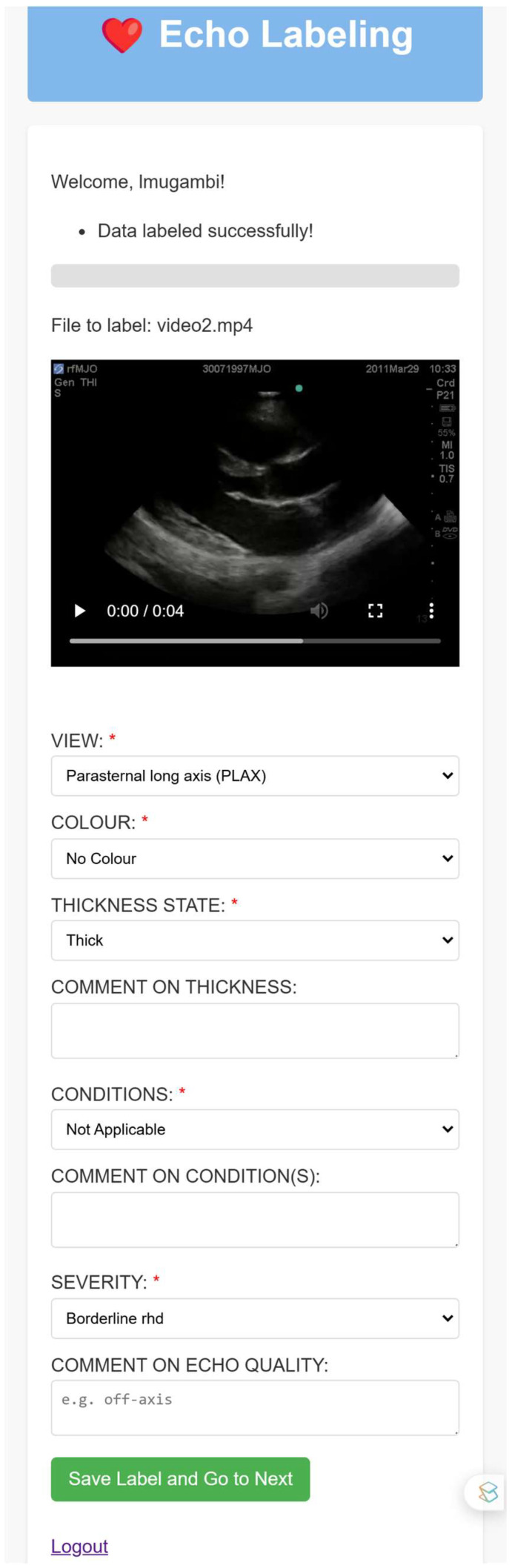
A screenshot the Echo Label application’s user interface, showing a video to be annotated, the metadata required for the video, a ‘save’ button, and a ‘logout’ option.

All these options are shown in a snippet of the application’s user interface (UI) in Figure 3 below.

- Progress Bar: a progress bar, as seen in Figure 3 above, was present to indicate the user’s annotation progress, motivating users to complete the labelling task.
- Database Integration: the application seamlessly integrates with a MySQL database, enabling the efficient storage, retrieval, and management of annotated data.

The “Not Applicable” label was employed when the specific condition or severity did not apply to the echocardiographic image being analysed. This may have occurred in cases where the image did not have sufficient information to classify a condition or when the heart’s anatomy appeared normal without any identifiable pathologies.

To ensure the accuracy of the labels, two qualified professionals were given access to the Echo Label application. The annotations from both professionals were cross-checked, and only the matching labels were used for the final dataset. This rigorous approach enhanced the reliability and quality of the labelled data. Each label assigned to a video was translated to the individual frames extracted from that video, ensuring that the temporal context of the echocardiographic data was preserved. The code for the Echo Label application is publicly available on GitHub at https://github.com/DeKUT-DSAIL/rhd-echo-label.git. During development and testing, the application was deployed on the Google Cloud Platform (GCP) App Engine, allowing multiple users to access it simultaneously.

**Figure 4:**
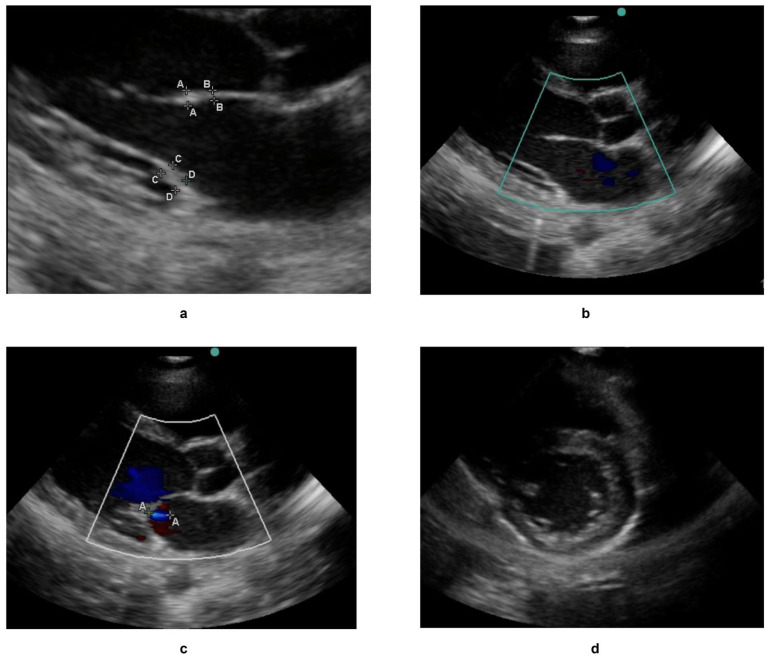
A sample of echocardiograms used as part of the labelled dataset, with the corresponding labels of all four images shown in Table 1. (**a**–**c**) are echocardiograms with a parasternal long axis view while (**d**) is an echo with a parasternal short axis view. The thickness state, RHD condition if any, and the severity of RHD condition if present have also been shown in Table 1. The uppercase letters, such as A–A or B–B denote standardized measurement points for structures such as valve thickness.

The large unlabelled subset was used to pre-train self-supervised models (SimCLR and DINOv2) to learn general representations of echocardiographic features. The labelled subset was then partitioned into training (70%), validation (15%), and test sets (15%) for supervised fine-tuning in order to detect and classify RHD-induced valvular pathologies. This combined approach—leveraging unlabelled data for pretraining and labelled data for task-specific refinement—ensured robust model performance. Sample images from the dataset are shown in Figure 4 and corresponding labels are shown in Table 1.

### 2.3. Data Preprocessing and Augmentation

Before model training, the dataset underwent several preprocessing steps:

1. Image Resizing: images were resized to a consistent size of [224 × 224] to ensure uniform input to the models.
2. Pixel Intensity Normalisation: pixel intensities were normalised to a range of 0–1 to improve model performance and prevent bias due to variations in image brightness.
3. Grayscale Conversion: images were converted to grayscale to reduce computational complexity and potentially improve model robustness to variations in colour.

Data augmentation techniques were applied to increase data diversity and mitigate potential overfitting. These techniques included:

1. Random cropping: randomly cropping image sections to create variations in the field of view.
2. Horizontal flipping: randomly flipping images horizontally to augment the dataset with mirrored versions.
3. Random rotations: rotating images by random angles within the range of [−15 to 15 degrees] to introduce rotational variations.
4. Brightness/contrast adjustments: adjusted brightness and contrast within the range of [−0.2 to 0.2] to simulate variations in imaging conditions.
5. Padding: to protect patient privacy when handling medical images, especially patient information around the images, padding was used. By padding the area containing patient information with a constant value, the aspect ratio of the images was maintained, and shearing was avoided.

### 2.4. Self-Supervised Learning

#### 2.4.1. SimCLR Implementation

Contrastive learning is a self-supervised technique that enables the extraction of meaningful representations from unlabelled data by contrasting similar and dissimilar instances. Among various methods, SimCLR excels in maximising agreement between augmented views of the same instance while minimising agreement for different instances. This is achieved through a large-batch training scheme and a neural network architecture, employing a contrastive loss to enhance training stability [27].

**Figure 5:**
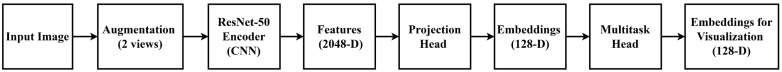
Architecture overview of the SimCLR model: (1) input echocardiogram; (2) augmentation generates two views; (3) ResNet-50 extracts hierarchical features using residual connections; (4) 2048-D feature vector; (5–6) projection head compresses features to 128-D; (7) task-specific classifiers; (8) embedding visualisation.

The ResNet-50 architecture (Figure 5, Block 3) is a convolutional neural network (CNN) that is widely used for image analysis. Its key innovation is “residual connections”—shortcuts that allow the network to bypass layers during training. This prevented the model from losing important details as it processes deeper into the image, making it highly effective for identifying hierarchical patterns in echocardiograms where:

- Early layers: detect edges and textures (e.g., heart chamber boundaries).
- Middle layers: recognize shapes (e.g., valves, myocardium).
- Final layers: capture complex spatial relationships such as blood flow patterns.

The output was a 2048-dimensional feature vector (Figure 5, Block 4) that summarizes the image’s visual content.

The contrastive loss function, the normalized temperature-scaled cross-entropy (NT-Xent) loss defined in Equation (1), was used. It compares positive pairs (augmented views of the same image) against negative pairs (views from different images) [30,31,32].

(1) $$L=-\sum \log\left(\frac{\exp\left(\frac{sim\left(x,\;x^{+}\right)}{\tau }\right)}{\exp\left(\frac{sim\left(x,\;x^{+}\right)}{\tau }\right)+\exp\left(\frac{sim\left(x,\;x^{-}\right)}{\tau }\right)}\right)$$

where x is the input image, $x^{+}$ is the positive view of the image, $x^{-}$ is the negative view of the input image, sim is the similarity function, and τ is the temperature parameter (0.07), and it is used to scale similarity scores. It was selected via a grid search (testing values between 0.05–0.5) to balance sensitivity to similarity differences.

After feature extraction, a projection head (Figure 5, Blocks 5–6), which was a small neural network with two fully connected layers and a ReLU activation function, was used to map the 2048-D features to a lower-dimensional space (128-D), optimized for contrastive learning. Finally, three task-specific linear classifiers (Figure 5, Block 7) leveraged these 128-D embeddings to predict:

- View classification (e.g., apical four chamber, parasternal long axis)
- RHD condition detection (e.g., mitral regurgitation, aortic stenosis)
- RHD severity assessment (e.g., borderline RHD/definite RHD)

#### 2.4.2. DINOv2 Implementation

**Figure 6:**
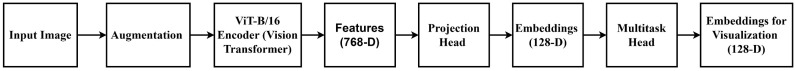
Architecture overview of the DINOv2 model: (1) input image; (2) patch splitting and positional encoding; (3) vision transformer (ViT) processes patches via self-attention; (4) 768D contextualized features; (5–8) similar to the SimCLR workflow, with frozen ViT during fine-tuning.

To leverage the power of vision transformers (ViTs) in DINOv2, the echocardiogram images were pre-processed by dividing them into smaller patches. Each patch was flattened into a 1D vector, and positional embeddings were added to encode its spatial information. The DINOv2 architecture consisted of the following components:

ViTs used in DINOv2 (Figure 6, Block 3), process images differently from CNNs. Instead of analysing pixels sequentially, the ViT:

- Divides the image into 16 × 16 patches (e.g., splitting a 224 × 224 image into 196 patches).
- Flattens each patch into a 1D vector and adds positional embeddings to retain spatial context.
- Processes patches through transformer layers that use self-attention mechanisms. Self-attention allows the model to weigh relationships between all patches simultaneously—for example, linking a valve patch to adjacent blood flow patterns. The output is a 768-dimensional feature vector (Figure 6, Block 4) encoding global image context.

DINOv2 trains two networks simultaneously for self-supervised learning:

- Student network: updated via gradient descent to predict the teacher’s output.
- Teacher network: a slowly updated version of the student (using exponential moving averages).

This self-distillation process forces the student to learn robust representations by matching the teacher’s predictions on unlabelled data, effectively “learning from itself”.

The teacher network generates pseudo-labels for the student network, which is trained to match the output distribution of the teacher network, enabling the model to learn meaningful representations without labelled data [28]. After pretraining, the ViT backbone is frozen to preserve learned features, and task-specific heads (Figure 6, Block 7) are trained on labelled data. This leverages both:

- General patterns from self-supervised pre-training (e.g., cardiac anatomy).
- Task-specific signals from fine-tuning (e.g., severity thresholds).

### 2.5. Multitask Learning

This refers to a situation where a shared encoder backbone simultaneously learns to perform several distinct but related tasks. In this case, three related cardiology tasks a cardiologist may use to diagnose RHD were considered. These are:

1. View classification: the model learns to identify three different echocardiographic views, i.e., the parasternal long axis, parasternal short axis, and apical four chamber. This ensures proper image acquisition angles in real life.
2. Condition detection: using the same encoded features, the model identifies various heart valve conditions indicative of RHD, such as mitral valve regurgitation and aortic valve regurgitation.
3. Severity assessment: the third task determines the severity levels of RHD. It could be borderline or definite RHD.

The advantages of this design are that low-level features benefit all tasks, and having multiple tasks improves model generalization and reduces overfitting [25,29,33,34]. The multitasking approach mirrors clinical practice, where cardiologists simultaneously assess view quality and conditions and then determine disease severity when examining echocardiograms.

After self-supervised pre-training, both models were fine-tuned on the labelled dataset. This involved training linear classifiers on the frozen pre-trained encoders for each task: view classification, condition detection, and severity assessment. This multi-task approach allowed each classifier/head to optimise for its objective, benefiting from the rich pre-trained features. The cross-entropy loss function (see Equation (2)) shown below was used for training.

(2) $$L=-\sum_{c=1}^{M}y_{c}\log{(p}_{c})$$

where:

- L is the cross-entropy loss;
- M is the number of classes;
- $y_{c}$ is the binary indicator (0 or 1) of whether class c is the true class for the sample;
- $p_{c}$ is the predicted probability that the sample belongs to class c.

### 2.6. Evaluation Metrics

The model’s performance was assessed using the following core metrics:

1. Accuracy: the performance of correctly classified images offering a general measure of predictive performance [29].
2. Precision: the ratio of true positive predictions to total predicted positives, reflecting the accuracy of positive classifications.
3. Recall: the ratio of true positive predictions to actual positives, indicating the model’s ability to detect all positive cases.
4. F1 Score: the harmonic mean of precision and recall, providing a balanced metric that is particularly valuable for imbalanced datasets [29].

In addition to the above-mentioned metrics, confusion matrices, which visualize prediction distributions across classes, as well as the ROC curve with its AUC, were generated to measure the model’s ability to discriminate between different classes in the three tasks considered [19]. These metrics collectively enabled a comprehensive evaluation of the models’ diagnostic accuracies for echocardiogram classification, revealing their strengths and limitations while assessing their potential clinical utility.

## 3. Results

This section details the results of training the SimCLR and DINOv2 models. Using a large echocardiogram dataset for pre-training and a smaller, labelled dataset for fine-tuning, the models’ ability to classify echocardiographic view, condition, and RHD severity was evaluated. Performance was measured using accuracy, precision, recall, and the F1 score. Both models showed significant improvement after fine-tuning. Feature visualisations using t-distributed Stochastic Neighbor Embedding (t-SNE) and Uniform Manifold Approximation and Projection (UMAP) revealed distinct clustering patterns for different classes.

### 3.1. Labelling Application

The Echo Label application was successfully implemented and tested with our consulting medical professional. The sample annotations in Table 1 demonstrate the application’s ability to capture metadata, which proved critical for fine-tuning. The dataset comprised 2655 labelled and 38,037 unlabelled images, encompassing various RHD-related views, conditions, and severities. The distribution of the annotations derived from the Echo Label application for all labelled data is shown in Table 2.

### 3.2. Classification

The classification pipeline began with data preparation. Both SimCLR and DINOv2 utilised the same dataset of echocardiogram images, which was divided into labelled and unlabelled subsets. The labelled set was further partitioned into training, validation, and test sets to facilitate model development and evaluation.

The next stage was self-supervised pre-training. SimCLR employs a contrastive learning approach, generating augmented views of each image using random transformations. A ResNet encoder and projection head were used to extract features and maximise the agreement between augmented views of the same image. In contrast, DINOv2 uses a teacher–student framework with self-distillation. Images were divided into patches, positional embeddings were added, and the images were processed using a vision transformer (ViT) backbone. The teacher network provided pseudo-labels for the student network, guiding the learning of meaningful representations.

Following pretraining, the pipeline proceeded to feature extraction and multi-task learning. In SimCLR, the pre-trained ResNet encoder extracted a feature vector from each input image. Then, separate linear classifiers were trained on these features for each task: view classification, condition detection, and severity assessment. With DINOv2, a pre-trained ViT backbone was used to extract features, employing a multi-task learning approach with a shared backbone and task-specific heads for each classification task.

For both models, the fine-tuning stage involved training classifiers (SimCLR: separate linear classifiers; DINOv2: task-specific heads) on the pre-trained representations to predict view, condition, and severity. As described in Section 2.4, a cross-entropy loss function was used for each task. Finally, the performance of both models was evaluated on the held-out test set using standard classification metrics (accuracy, precision, recall, and the F1 score). To provide further insight into the learnt representations, confusion matrices and visualisations using t-SNE and UMAP were generated (Appendix B and Appendix C).

### 3.3. Performance Metrics Comparison

Table 3 summarises the performance metrics achieved by both models on the test set for the three classification tasks.

### 3.4. Embeddings Visualisation

A critical aspect of evaluating the effectiveness of self-supervised learning methods is to assess the quality of the learned representations. To achieve this, we employed dimensionality-reduction techniques—t-SNE and UMAP [35,36,37]—to visualize the high-dimensional embeddings generated. These techniques project the embeddings into a two-dimensional space, allowing us to examine the resulting clustering patterns visually. UMAP works by preserving both the local and global structure of the data by approximating the underlying manifold structure, while t-SNE focuses on preserving the local structure of the data by mapping similar data points close together and keeping dissimilar points far apart. UMAP is often faster and scales better to large datasets compared to t-SNE while also providing a better balance of local and global structure preservation [37,38,39,40]. If the models have learned to extract meaningful, discriminative features, we can expect to see compact and well-separated clusters corresponding to each class (i.e., different echocardiographic views, RHD conditions, and severity levels). The presence of such clusters would indicate that the models have successfully captured the underlying semantic relationships between echocardiogram images.

**Figure 7:**
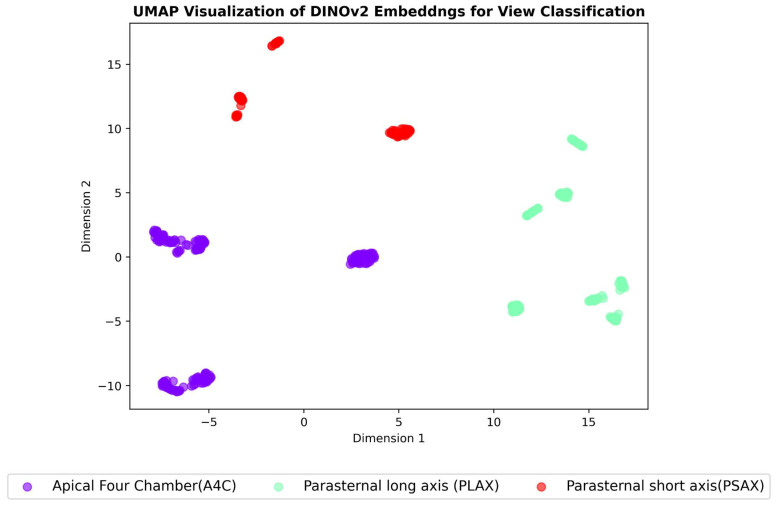
UMAP visualisation of DINOv2 embeddings for view classification. Each point represents an embedding, with colours indicating the corresponding view class.

**Figure 8:**
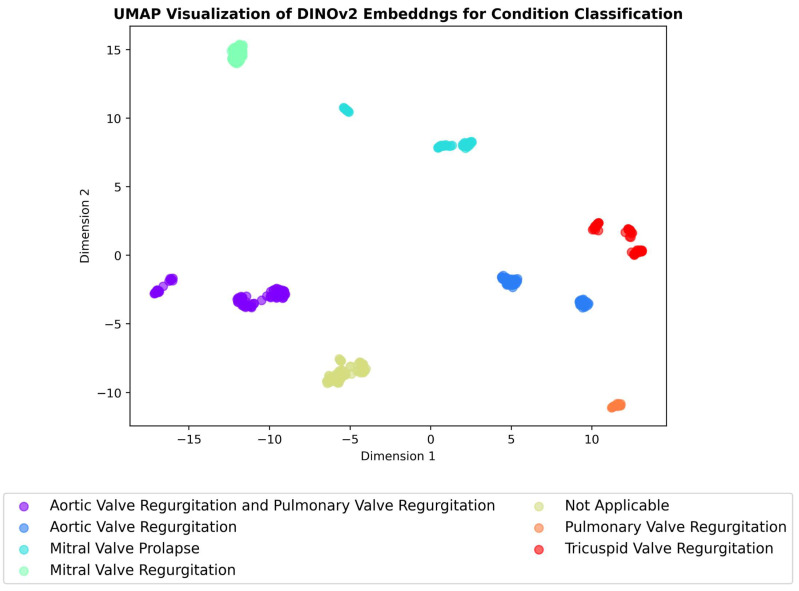
UMAP visualisation of DINOv2 embeddings for condition detection. Each point represents an embedding, with colours indicating the corresponding condition class.

**Figure 9:**
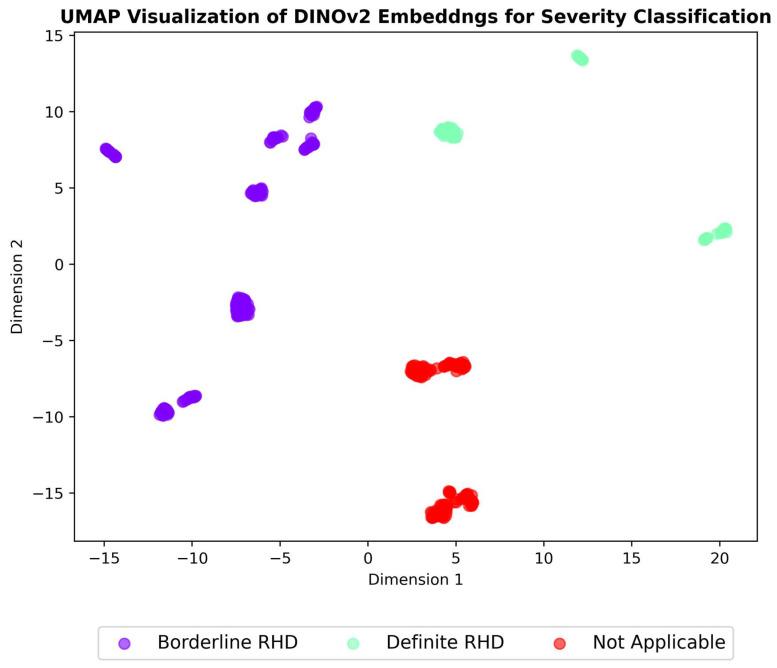
UMAP visualisation of DINOv2 embeddings for severity assessments. Each point represents an embedding, with colours indicating the corresponding severity class.

The results of these visualisations are shown in Figure 7, Figure 8 and Figure 9, which illustrate the UMAP-reduced DINOv2 embeddings for view classification, condition diagnosis, and severity assessment, respectively. The figures depict distinct and compact clusters for each class, suggesting that DINOv2 has learned representations that are highly effective for the three classification tasks. These observations are further supported by the t-SNE visualisations included in Appendix C (Figure A5, Figure A6 and Figure A7), which demonstrate consistent clustering patterns.

**Figure A2:**
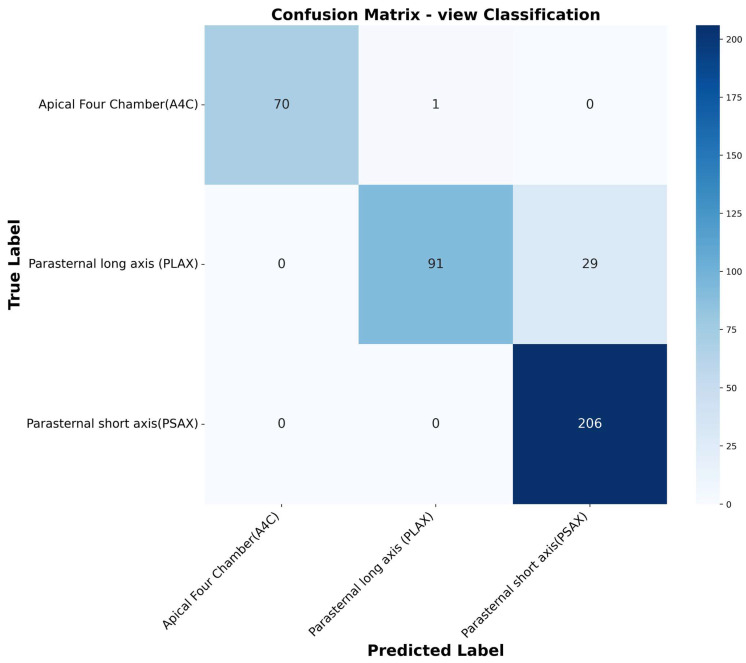
Confusion matrix for view classification.

**Figure A3:**
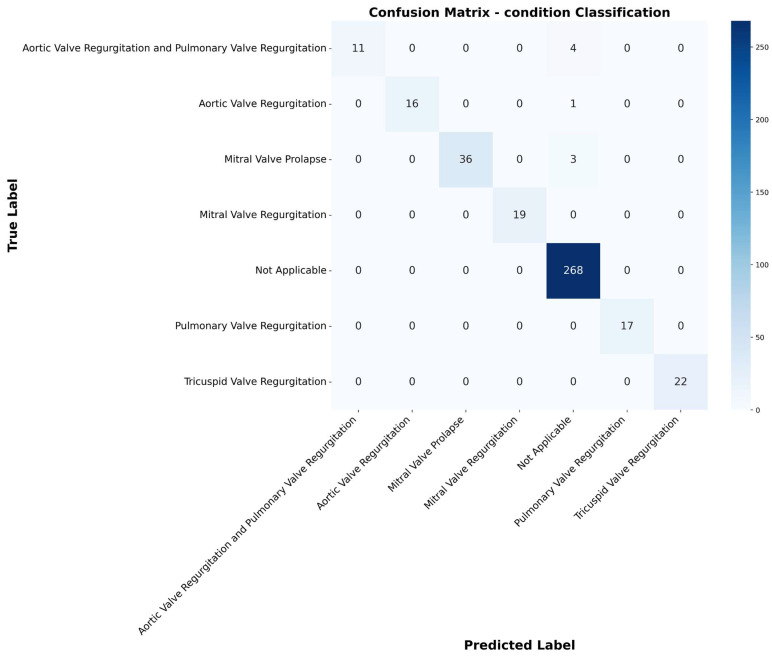
Confusion matrix for condition detection.

**Figure A4:**
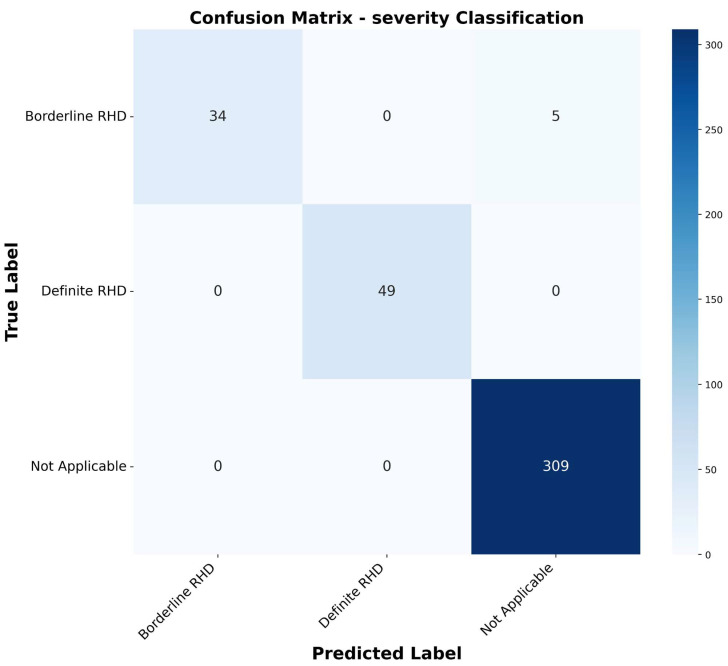
Confusion matrix for severity assessment.

**Figure A5:**
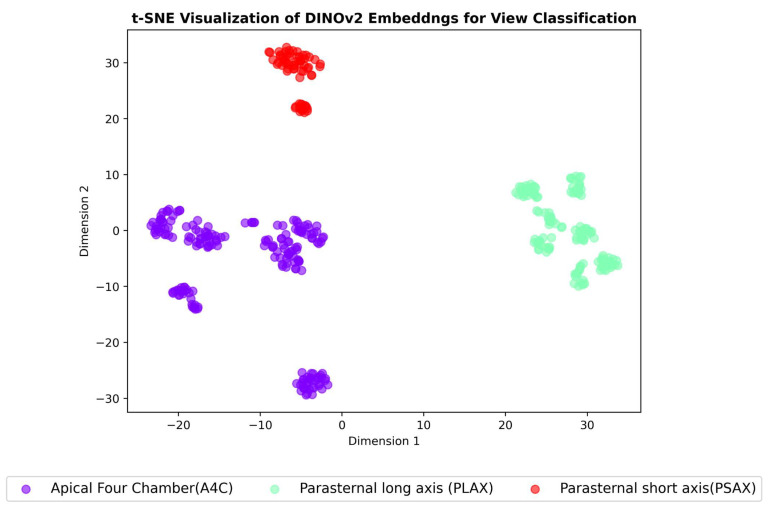
t-SNE Embeddings for view classification—DINOv2.

**Figure A6:**
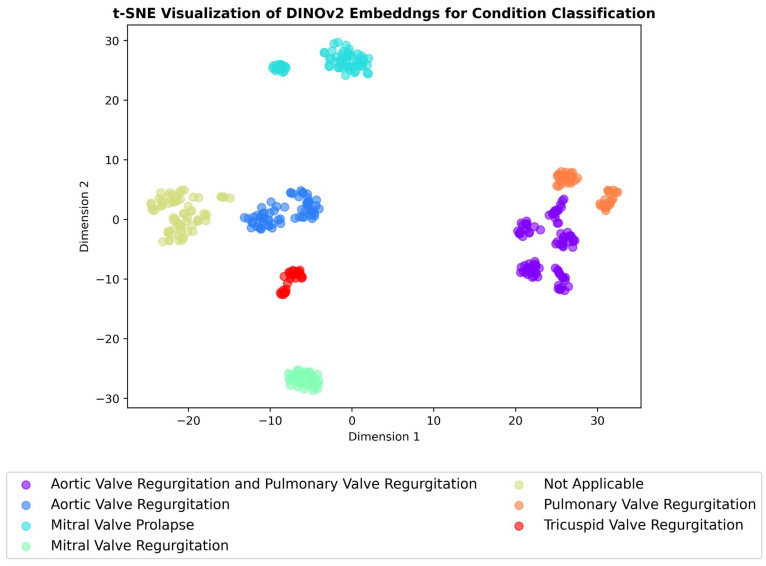
t-SNE Embeddings for condition classification—DINOv2.

**Figure A7:**
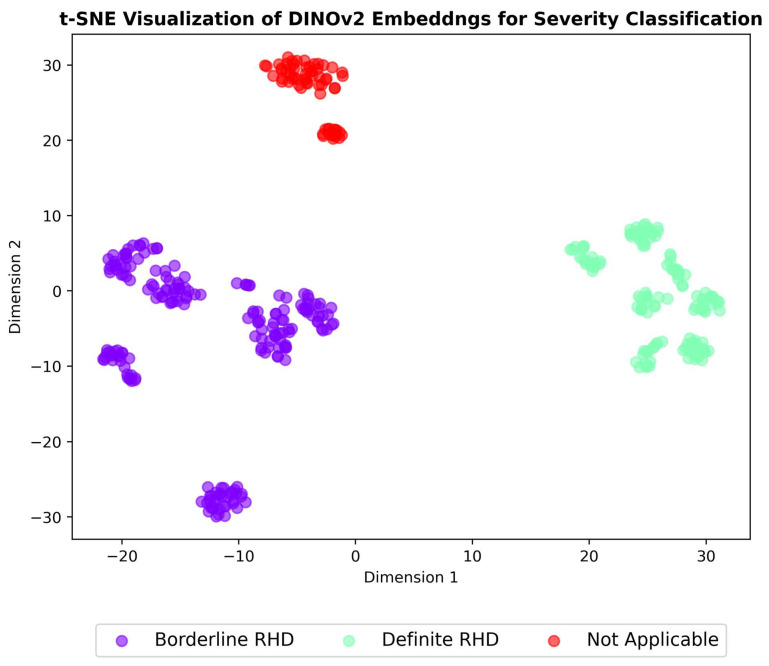
t-SNE Embeddings for severity assessment—DINOv2.

**Figure A8:**
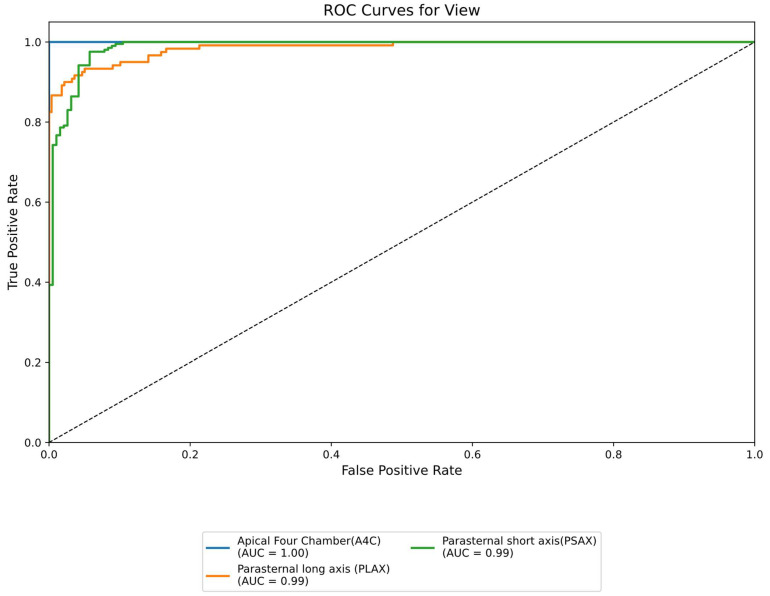
ROC curves and AUC for view classification.

**Figure A9:**
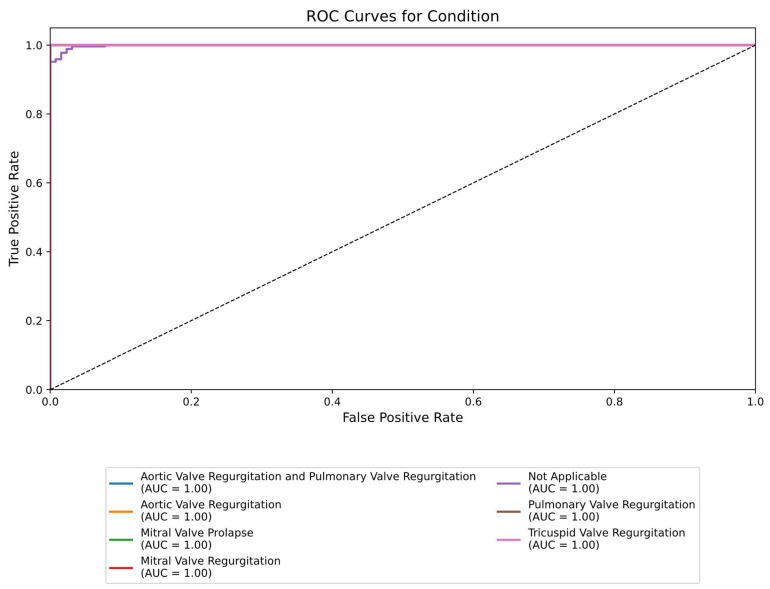
ROC curves and AUC for condition detection.

**Figure A10:**
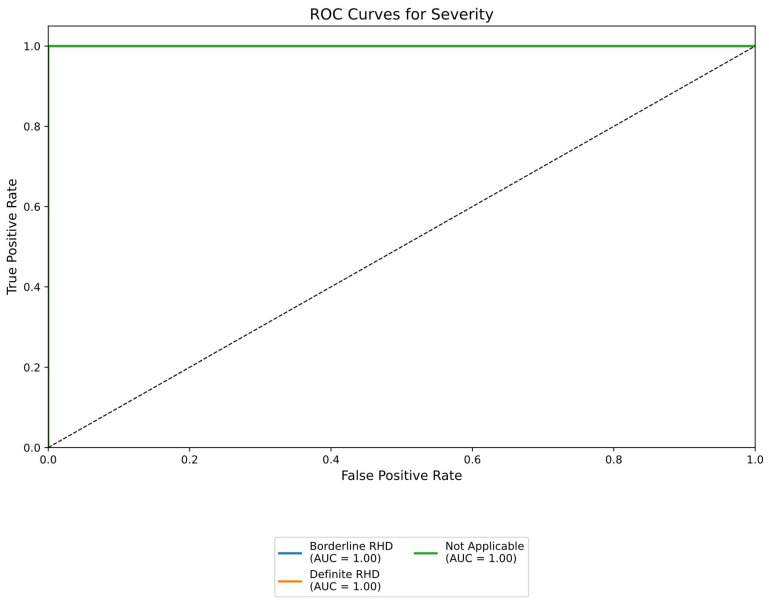
ROC curves and AUC for severity classification.

To complement this high-level visualization of the learned representations, we present a more granular analysis of the DINOv2 model’s classification performance through confusion matrices for each task (view identification, RHD condition diagnosis, and RHD severity assessment) in Appendix B (Figure A2, Figure A3 and Figure A4), as well as the AUC values in Appendix D (Figure A8, Figure A9 and Figure A10). These matrices provided insight into classification accuracy and error types, highlighting the model’s strengths and weaknesses. Unlike in manual analyses, our automated system required the model to infer the view, as it is not known beforehand. Accurate view identification is essential for downstream tasks, and so the view confusion matrix was particularly significant. This is because different views showed different structures, and the models’ ability to generalize to unseen data was key.

## 4. Discussion

This study demonstrated the efficacy of using self-supervised multi-task learning for automated echocardiogram analysis, specifically targeting RHD detection and classification. Both SimCLR and DINOv2 achieved high performance across view classification, condition detection, and severity assessment tasks, highlighting the potential of SSL in medical imaging. The results, supported by robust evaluation metrics, provide valuable insights into the strengths and limitations of these models.

SimCLR achieved exceptional performance in view classification, with accuracy of 0.99, precision of 0.99, recall of 0.99, and an F1 score of 0.99. This outperformed DINOv2, which achieved accuracy of 0.92, precision of 0.93, recall of 0.92, and an F1 score of 0.92. The superior performance of SimCLR in this task may be attributed to its contrastive learning framework, which excels at learning invariant features under different augmentations, a critical requirement for distinguishing between echocardiogram views. Visualizations of the learned feature spaces using UMAP (Figure 7, Figure 8 and Figure 9) further support this by showing tighter and more separable clusters for SimCLR than for DINOv2.

For condition detection, DINOv2 outperformed SimCLR, achieving accuracy of 0.98, precision of 0.98, recall of 0.98, and an F1 score of 0.98, compared to SimCLR’s accuracy of 0.92, precision of 0.92, recall of 0.92, and F1 score of 0.92. This suggests that DINOv2’s architecture, which leverages richer feature representations, is better suited for capturing discriminative features in condition classification tasks.

In the severity assessment, DINOv2 also demonstrated superior performance, achieving accuracy of 0.99, precision of 0.98, recall of 0.98, and an F1 score of 0.99, compared to SimCLR’s accuracy of 0.96, precision of 0.97, recall of 0.97, and F1 score of 0.96. The near-perfect AUC scores of 1 for all classes in both the condition and severity tasks further underscore DINOv2’s robustness in these tasks. This indicates that DINOv2’s ability to extract more nuanced features makes it particularly effective for fine-grained severity classification.

Notably, the borderline RHD cases in Figure 9 decompose into two subclasses, which may reflect variations in echocardiogram features such as valve morphology or Doppler patterns. While these clusters provide valuable insights into the learned feature space, further analysis is needed to fully interpret their clinical significance. While the UMAP provides a high-level view, a more in-depth analysis of the original video frames is necessary to fully interpret these clusters.

Our findings align with recent studies demonstrating the effectiveness of SSL in medical imaging tasks. For instance, Holste et. al. and Sangha et. al. [41,42] have shown that SSL models such as SimCLR and DINOv2 can achieve state-of-the-art performance with limited labelled data. However, unlike prior works that focus on single-task learning, our multi-task framework achieved competitive performance across multiple tasks simultaneously. This suggests that multi-task learning can effectively leverage shared features across tasks, particularly in data-constrained settings.

A key strength of this study is the use of SSL for multi-task learning, which enabled accurate classification with limited labelled data. The inclusion of the “Not Applicable” label enhanced real-world applicability by addressing challenging cases, while the Echo Label application streamlined data annotation and improved dataset quality. However, the study has limitations. The dataset, while substantial, is limited in size and diversity, consisting primarily of school-going children in South Africa. Expanding the dataset to include diverse age groups, clinical conditions, and geographic regions would improve generalizability. While our preprocessing pipeline addressed many challenges, the original echocardiogram images were of varying quality due to the nature of the clinical environment in which they were acquired. Future work could explore advanced denoising methods, such as to further improve image quality and robustness. Additionally, while the models performed well, further analysis of the learned feature representations (e.g., feature distribution comparisons) could provide deeper insights into their behaviour.

The computational cost of the experiments was evaluated to assess the feasibility of the proposed methods for real-world deployment. All experiments were conducted using 2 NVIDIA T4 GPUs with mixed-precision training to optimize efficiency. For SimCLR pretraining, 250 epochs required approximately 24 h. For DINOv2, the pretraining and fine-tuning pipeline for 250 epochs required approximately 21 h. These results demonstrate that the proposed methods are computationally feasible, particularly given the high performance achieved with relatively modest training times. The use of mixed-precision training and multi-GPU parallelism significantly reduced the computational burden, making the approach suitable for real-world deployment.

Future research will focus on expanding the dataset to include more diverse populations, exploring explainable AI techniques to improve model interpretability, and investigating alternative architectures and multi-task learning strategies. Combining images with other multimodal signals such as sound or ECGs to improve the accuracy and reliability of such a system is also an interesting research area we hope to explore. Collaborations with public health organizations and NGOs could facilitate the deployment of this model in resource-limited settings, while clinical validation and pilot studies will be essential to assess its real-world impact on RHD diagnosis and patient outcomes.

## 5. Conclusions

This study demonstrated the potential of using self-supervised multi-task learning for automated echocardiogram analysis, offering a promising avenue for RHD diagnosis. SimCLR achieved exceptional performance in view classification, with accuracy of 0.99, precision of 0.99, recall of 0.99, and an F1 score of 0.99, outperforming DINOv2 in this task. For condition detection, DINOv2 achieved superior performance, with accuracy of 0.98, precision of 0.98, recall of 0.98, and an F1 score of 0.98, compared to SimCLR’s accuracy of 0.92. In the severity assessment, DINOv2 also outperformed SimCLR, achieving accuracy of 0.99, precision of 0.98, recall of 0.98, and an F1 score of 0.99, compared to SimCLR’s accuracy of 0.96. The near-perfect AUC scores of 1 for all classes in condition and severity tasks further highlight DINOv2’s robustness.

The embedding visualizations using t-SNE and UMAP confirmed that both models learned meaningful and discriminative features, with SimCLR showing superior feature separation in view classification. The multi-task learning framework enabled effective knowledge transfer across tasks, resulting in high performance despite the limited labelled data. The Echo Label application played a critical role in ensuring high-quality data annotation, contributing to the study’s success.

These findings underscore the potential of SSL and multi-task learning to overcome data limitations in medical imaging. However, further research is needed to validate these results on larger and more diverse datasets, explore model interpretability, and assess real-world applicability. By addressing these challenges, this approach could significantly improve RHD diagnosis and patient outcomes, particularly in resource-limited settings.

## Figures and Tables

**Table 1:** Sample annotations from the Echo Label application, showing the captured metadata for various echocardiogram files.

	Filename	Colour	Echo View	Thickness	Condition	Severity
a	10.38.47 hrs __[0005903].jpeg	NO COLOUR	Parasternal long axis (PLAX)	Thick	Not applicable	Definite RHD
b	10.39.22 hrs __[0005905].mp4	COLOUR	Parasternal long axis (PLAX)	Not thick	Aortic valve regurgitation	Borderline RHD
c	10.40.10 hrs __[0005906].jpeg	COLOUR	Parasternal long axis (PLAX)	Not applicable	Mitral valve regurgitation	Normal
d	10.40.15 hrs __[0005907].mp4	NO COLOUR	Parasternal short axis (PSAX)	Not applicable	Mitral valve prolapse	Not applicable

**Table 2:** Distribution of samples across different classes for view, condition, and severity labels in the labelled dataset.

View Classification		
**Class**	**Numerical Encoding**	**Number of Samples**
Apical Four Chamber (A4C)	0	483
Parasternal Long Axis (PLAX)	1	724
Parasternal Short Axis (PSAX)	2	1448
**Condition Classification**		
**Class**	**Numerical Encoding**	**Number of Samples**
Aortic Valve Regurgitation	0	121
Aortic Valve Regurgitation, Pulmonary Valve Regurgitation	1	121
Mitral Valve Prolapse	2	241
Mitral Valve Regurgitation	3	121
Not Applicable	4	1809
Pulmonary Valve Regurgitation	5	121
Tricuspid Valve Regurgitation	6	121
**Severity Classification**		
**Class**	**Numerical Encoding**	**Number of Samples**
Borderline RHD	0	242
Definite RHD	1	241
Not Applicable	2	2172

**Table 3:** Performance metrics (accuracy, precision, recall, and F1-score) for the SimCLR and DINOv2 models on the test set for each classification task.

Metric	Task	SimCLR	DINOv2
Accuracy	View	**0.99**	0.92
	Condition	0.92	**0.98**
	Severity	0.96	**0.99**
Precision	View	**0.99**	0.93
	Condition	0.92	**0.98**
	Severity	0.97	**0.98**
Recall	View	**0.99**	0.92
	Condition	0.92	**0.98**
	Severity	0.97	**0.98**
F1 score	View	**0.99**	0.92
	Condition	0.92	**0.98**
	Severity	0.96	**0.99**

**Figure A1:**
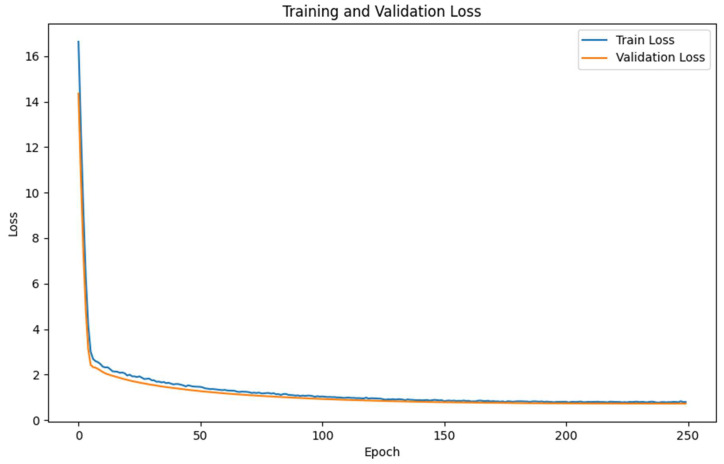
Training and validation loss curves.

## Data Availability

Due to the sensitive nature of medical data and patient privacy concerns, the dataset used in this study cannot be made publicly available.

## Appendix B

To further assess the DINOv2 model’s performance on each classification task—view identification (Figure A2), RHD condition diagnosis (Figure A3), and RHD severity assessment (Figure A4)—we present the corresponding confusion matrices. These matrices offered a detailed breakdown of the model’s ability to distinguish between each class, highlighting areas of strength and areas where misclassification occurred. The confusion matrices provided counts of true positives, false positives, and false negatives, allowing for an in-depth error analysis that is crucial for understanding the model’s limitations and potential clinical implications.

## Appendix C

This section presents the t-SNE visualisations of the embeddings learnt by the models. These visualisations provide a complementary perspective to the UMAP embeddings presented in the main text, focusing on the local structure and separation of clusters.

## Appendix D

This section shows the ROC curves and AUC values for view classification, condition detection, and severity assessment from DINOv2.
